# Patient and health professionals’ perspectives on the use of ciclosporin and infliximab when treating acute severe ulcerative colitis: an added dimension to the construct trial

**DOI:** 10.1186/1745-6215-16-S2-P72

**Published:** 2015-11-16

**Authors:** Clare Clement, Frances Rapport

**Affiliations:** 1 Swansea University, Swansea, UK

## Background

The efficacy of Infliximab and Ciclosporin for treating moderate to severe ulcerative colitis is proven, but their relative effectiveness has not been evaluated. There is also limited understanding of patients’ and professionals’ views of the drugs.

## Aim

The COmparison Of iNfliximab and ciclosporin in STeroid Resistant Ulcerative Colitis Trial (CONSTRUCT) aimed to compare the clinical and cost effectiveness of infliximab and ciclosporin in the treatment of steroid resistant acute severe ulcerative colitis, using quantitative, qualitative, and health economic data. The qualitative element explored patients’ and health professionals’ views of the two drugs.

## Method

Within a UK multi-centre open label pragmatic randomised controlled trial, 35 interviews were conducted with 20 patients and 23 health professionals involved in the trial.

## Findings

There was no significant difference between the two drugs in clinical effectiveness terms, incidence of serious side effects, colectomy rates or mortality. Overall, service costs were significantly greater with infliximab, but it was viewed more positively by patients and nurses, who disliked the inconvenience of ciclosporin infusions. Healthcare professionals described the need for nurses with special knowledge of using ciclosporin for ease of drug handling.

## Conclusion

Integrated interview data with other datasets allowed us to bring additional insights about the impact of treatment and subjective benefits and disbenefits of the drugs to this trial. We could also describe context-specific preferences for the drugs from those directly responsible for patient care, offering an added dimension to clarifying decision-making in patient care, thus enabling a more holistic approach to interventional drug treatments.

